# Eye-brain axis in microgravity and its implications for Spaceflight Associated Neuro-ocular Syndrome

**DOI:** 10.1038/s41526-023-00300-4

**Published:** 2023-07-20

**Authors:** Claudia Stern, Yeni H. Yücel, Peter zu Eulenburg, Anne Pavy-Le Traon, Lonnie Grove Petersen

**Affiliations:** 1 Institute of Aerospace Medicine, German Aerospace Center (DLR), Cologne, Germany; 2 ISS Operations and Astronauts Group, European Astronaut Centre, European Space Agency (ESA), Cologne, Germany; 3 Keenan Research Centre for Biomedical Science, St. Michael’s Hospital, Unity Health Toronto, Toronto, ON Canada; 4 Department of Ophthalmology & Vision Sciences, Temerty Faculty of Medicine, University of Toronto, Toronto, ON Canada; 5 Department of Laboratory Medicine & Pathobiology, Temerty Faculty of Medicine, University of Toronto, Toronto, ON Canada; 6 Department of Physics, Faculty of Science, Toronto Metropolitan University, Toronto, ON Canada; 7 Department of Ophthalmology and Visual Sciences, Faculty of Medicine, University of British Columbia, Vancouver, BC Canada; 8 Institute for Neuroradiology & German Center for Vertigo and Balance Disorders, Ludwig-Maximilians-University, Munich, Germany; 9 Department of Neurology, University Hospital of Toulouse, Toulouse, France; 10 MEDES, Institute for Space Physiology and Medicine, Toulouse, France; 11 UMR INSERM U1297, Institute of Cardiovascular and Metabolic Diseases (I2MC), Toulouse, France; 12 Department of Aeronautics and Astronautics, Massachusetts Institute of Technology, Cambridge, USA; 13 Department of Biomedical Sciences, University of Copenhagen, Copenhagen, Denmark

**Keywords:** Translational research, Brain imaging

## Abstract

Long-duration human spaceflight can lead to changes in both the eye and the brain, which have been referred to as Spaceflight Associated Neuro-ocular Syndrome (SANS). These changes may manifest as a constellation of symptoms, which can include optic disc edema, optic nerve sheath distension, choroidal folds, globe flattening, hyperopic shift, and cotton wool spots. Although the underpinning mechanisms for SANS are not yet known, contributors may include intracranial interstitial fluid accumulation following microgravity induced headward fluid shift. Development and validation of SANS countermeasures contribute to our understanding of etiology and accelerate new technology including exercise modalities, Lower Body Negative Pressure suits, venous thigh cuffs, and Impedance Threshold Devices. However, significant knowledge gaps remain including biomarkers, a full set of countermeasures and/or treatment regimes, and finally reliable ground based analogs to accelerate the research. This review from the European Space Agency SANS expert group summarizes past research and current knowledge on SANS, potential countermeasures, and key knowledge gaps, to further our understanding, prevention, and treatment of SANS both during human spaceflight and future extraterrestrial surface exploration.

## Introduction

Anecdotes of changes in visual acuity associated with weightlessness have circulated since the early days of spaceflight, even prompting crew to bring along “anticipation glasses'' for longer missions to be able to maintain near-sight acuity months into flight. The etiology of SANS and its potential link with any structural brain changes remain unclear and underinvestigated^1,2^. Suggestions of elevated intracranial pressure (ICP) as a consequence of weightlessness induced headward fluid-shift were discussed as early as the 80s but were originally not considered a major risk. However, with increasing mission length, the severity of both ocular and brain-structural changes and resulting clinical symptoms have become more prevalent and SANS and SANS-related changes are now classified as a critical risk both for individual crew and overall mission integrity^3,4^. The Spaceflight Associated Neuro-ocular Syndrome (SANS) findings include globe flattening, hyperopic refractive error shift, optic nerve sheath distension, optic disc edema, choroidal and retinal folds and focal areas of ischemic retina (i.e., cotton wool spots)^5^. Brain health as a research focus has, for a large part, been ignored and lingered at the bottom of the priority-list of organ-systems investigated for changes due to exposure of microgravity. The research of the central nervous system over the past 5 years was not driven by symptoms reported by the astronauts (though space headache was mentioned by some), but rather by scientific curiosity to observe neuroplasticity in light of the otherworldly and unique living experience. The changing role for the vestibular system and the lack of locomotion in space were obvious topics as well as the consequences of the confinement and social isolation aboard a space station.

Commonly ophthalmologists cover the topic of eye changes, while neurophysiologists and neurologists cover cerebral and functional changes associated with spaceflight. Finally, flight surgeons handle medical issues either acutely arisen or when/if adaptation changes cross the pre-clinical threshold to cause symptoms and decreased performance. This paper combines various backgrounds, experience and expertise with interdisciplinary perspectives facilitated by ESA’s SciSpacE White Papers (12_HumanResearch_HumanPhysiology.pdf(esa.int)) initiative. It connects knowledge from scientific and operational areas and provides input for future research and development (Table 1). In this article no new data were created or analyzed and therefore no written informed consent or granted approval were required.

**Table 1:** SANS components and occurrence in space and analogs according to the current definition of SANS.

	Space	Head-down tilt bed rest	Dry immersion	Isolation and confinement
Optic disc edema	Yes	Yes	No	No
Optic nerve sheath distension	Yes	Yes	Yes	No
Choroidal and retinal folds	Yes	Yes	No	No
Globe flattening and hyperopic shift	Yes	No	?	No
Cotton wool spots	Yes	No	No	No

SANS pathologies, that are seen in astronauts, could also be demonstrated in analog studies, especially head down tilt bed rest, but globe flattening and hyperopic shift, as well as cotton wool spots, have not been described in analog studies.

## Fluids

Due to globe flattening, choroidal folds, increase in choroidal thickness and optic nerve head protrusion in SANS, it is hypothesized that weightlessness causes a disproportionate elevation in ICP relative to IOP and thus reduction in the translaminar pressure gradient across the posterior part of the eye. Possible sources of entry of the fluid in the optic nerve head include the optic nerve capillaries, peripapillary choroid, vitreous^6^ and cerebrospinal fluid via the optic nerve glymphatic system^7^. In elevated IOP conditions such as in glaucoma the initial injury occurs in retinal ganglion cell axons at the lamina cribrosa of the optic nerve head^8–11^. IOP is also an integral part of the translaminar pressure difference that has been proposed to be involved in glaucoma pathogenesis^12^. Studies of IOP relative to gravitational stress and weightlessness are of great interest given the immediate increase in IOP noted upon entering weightlessness^6,8–10^. IOP has been reported to return to baseline values within the first week of microgravity exposure^9,10^. After return from long-duration spaceflight, IOP values were similar to preflight measures (10–14 mmHg versus 10–16 mmHg, respectively)^3^. It is important to note that IOP measured after landing on Earth may be lower than preflight levels. A postflight decrease of IOP compared with preflight measurements was already observed in Apollo astronauts^11^. IOP depends on both ocular hydrodynamics and hemodynamics^12,13^, and its measurements can inform us about both of these highly regulated systems. Although changes in IOP are not included in the definition of SANS, IOP and its hydro- and hemodynamics-determinants may be fundamentally relevant to our understanding and prevention of SANS^14^. The interstitial fluid in the retrobulbar optic nerve within the orbit is influenced by cerebrospinal fluid pressure (CSFP). The normal translaminar pressure (TLP) gradient will be affected by changes in IOP and/or ICP. In gravity conditions, TLP depends on the hydrostatic effects related to changes in body posture. Simultaneous measurements of IOP and ICP determined the TLP changes with subjects’ position changes in the short-term^15^. In nonhuman primates, TLP change due to body position change is driven more by ICP/CSFP than IOP^16^. However, the long-term effects of body position changes and of reduced gravity on TLP are not yet known. Simultaneous measurements of IOP and ICP along with jugular veins in ground analogs would be helpful. In addition to posture-related changes, the effects of circadian rhythm on the TLP components should be evaluated.

However, experimental data from parabolic flights measuring ICP directly demonstrated no increase in ICP, but rather an early decrease upon exposure to microgravity compared to supine posture.

The lymphatic and brain-specific glymphatic circulation have recently been established as a fluid removal system for both the eye^17^ and the central nervous system and are important areas of opportunity to address with SANS. With changes in gravitational forces, lymphatic drainage into the nasal^18^, meningeal^19^ and cervical lymphatics may be impaired due to changes in pumping ability^19,20^, passive flow due to stasis in major veins of the neck and increased venous pressure at the level of the jugular veins^19–21^. Further investigations are needed as a platform with which to consider therapeutic interventions. Lymphatics / glymphatic drainage and venous drainage from the eye and brain overlap with cardiovascular physiology and may present opportunities to increase understanding of normal human physiology and development of non-invasive quantitative tools to determine the lymphatic and cardiovascular changes simultaneously to better understand SANS. The quantitative approaches with non-invasive tests may support or substitute the OCT in early diagnosis of SANS and aid in determining pathophysiology.

Moreover, lack of habitual diurnal variability from daily posture changes as we stand up, sit and lay down, is lost in space. Astronauts cannot “stand up in space,”^22^ which is a major stress for SANS, and probably also the brain, because this likely represents a low, but continuous overload without any diurnal relief.

The pulsation of the (intracranial) vessels as well as their stiffness seem to have an influence on fluid drainage. Fluid shift also affects the interstitial tissue and the intrathoracic pressure is important in the distribution and drainage of the fluid. Low-dose radiation can damage the endothelial cells which also may increase the permeability of the lymphatic endothelial cells for fluid into the interstitial tissue^23^.

Transcranial ultrasonography has been widely used as a non-invasive real-time approach to determine changes in Cerebral Blood Flow (CBF) and Cerebral Autoregulation (CA) in spaceflight or in analogs. Either in spaceflights or in ground-based models of microgravity, most short-term studies have shown a preserved or even an improved CA. However, some long-term studies have depicted an impairment in CA^24^. In particular, an impairment of cerebral blood flow regulation has been reported in astronauts with orthostatic intolerance after flight^24,25^. Altered CA may also contribute to SANS.

In addition, cerebral circulation may be affected differently within the brain. An ultrasound study in long-term head-down bed rest (HDBR) has shown heterogeneous CBF changes between the anterior and posterior cerebral circulation^26^. Five days of dry immersion induced a decrease in regional CBF (measured by single-photon emission computed tomography (SPECT)—HMPAO) in cortical and subcortical regions^27^.

SANS was originally believed to be more prevalent in male crew because there were fewer female astronauts and only one reported female astronaut case^28^.

In most of the cases the right eye of the astronauts is more affected than the left eye^3,28^. This might relate to a slightly different anatomy and a potential increased pressure on the left common carotid artery by the left branchocephalic vein. Therefore, the amount of arterial blood flow into the brain can be reduced, which would result in a lower fluid accumulation compared to the right side. This idea is supported by a stagnant or reverse flow in the left internal jugular vein (IJV) and an occlusive IJV thrombus, and a potential partial IJV thrombus that were identified in crew members^21^.

Cerebral changes may differ between first-time and frequent flyers. The role of age with respect to the brain changes is unclear to date, but a stiffness of vessels with increasing age may hinder the fluid drainage.

Lumbar puncture opening pressure has been determined in several astronauts with optic disc edema postflight, but not preflight. Opening pressures in four astronauts have been published. In one case, the opening pressure was normal (21 cmH_2_O) considering a normal intracranial pressure of 6–25 cmH_2_0^29^, in the other cases it was measured mildly elevated between 26 and 28.5 cmH_2_O^3^. Because no preflight data was available, limited conclusions can be drawn. Efforts are ongoing to measure ICP (LP) pre-, in-, and post-flight; however, the value of a single measurement is questionable and might be uninformative with respect to the dynamic mechanism of cephalad fluid shift. To undertake lumbar puncture in healthy astronauts’ preflight raises ethical questions because of the risk-benefit consideration. This data from a single pressure value in time might even obscure rather than clarify the picture. We advocate the hypothesis that the lack of circadian variability in intracranial flow and pressure patterns might actually be part of the underpinning mechanisms of SANS and thus entirely be missed by single-point measurements^22,29,30^. The optimal site and time point of such a pressure recording, in addition to the length of the recording, is also deemed essential. Spinal transducer recordings at lumbar level, which appear to be considered for implementation, will not reflect intracranial pressure at the level of the cerebral ventricles and are therefore considered obsolete in neurosurgery^31^. We can also extrapolate from the present literature that no fundamental and lasting ocular and brain-structural alterations occur within the first 4–6 weeks in microgravity. So dynamic intracranial pressure data acquisition probably needs to be performed at baseline on Earth and during a long-duration mission of six months (e.g., at month 3 or 4).

To summarize, prolonged dynamic (continuous) measurements of all compartments of cranial (cerebral and ocular) circulation and 24-h assessments pre-, in-, and directly post-flight with respect to actual fluid turnover are therefore recommended to provide better insight into the underlying mechanisms and recovery process of SANS, SANS-associated changes, and brain-structural alterations.

Additional challenges relating to methodology include an inherently small sample size, which limits the ability to stratify for gender, age and other factors. Options for pooling data across specific trials, research groups, agencies, and crew-population are challenged by varying methods of data collection and variable timepoints of data collection. Emphasis on data sharing, including raw-data, and inclusion of all available data into computational models is strongly recommended.

Structural magnetic resonance imaging (sMRI) in a 3 T environment using mainly T1-weighted acquisitions has been shown to be the main workhorse to decipher brain changes in humans after long-duration exposure to microgravity (>3 months mission length). Slow intracranial expansion of the cerebrospinal fluid spaces over mission duration with a concurrent reduction in gray matter volume has been shown in cohorts from the American and Russian space agencies alike^32–34^. Similar to SANS, the starting point of cerebrospinal fluid accumulation is unclear. Comparisons with Shuttle missions argue for an onset after 4–6 weeks in microgravity. But all available studies in this regard are hampered by the delayed MRI data acquisition for the brain 4–10 days after return to Earth and may severely underestimate the actual intracranial fluid accumulation at the end of a long-duration mission. The longest follow-up sMRI data as of this writing is recorded half a year after return, still showing remarkable changes for all three brain tissue compartments. Basic harmonization of core mandatory neuroimaging protocols would be a big step forward to make cohorts comparable between space agencies and analogs while at the same time allowing for differing return sites. With the first description of two left-sided jugular vein thrombosis in astronauts^21,35^, MRI sequences to study intracranial blood flow will most likely be necessary to investigate the mechanism and scope of venous stasis for the entire head. All neuroimaging in small cohorts such as spaceflight and space analogs research should be aware of the time of day effect for quantifying structural changes. This means subjects should be scanned in a 4 h window similar to their baseline acquisition in longitudinal measurements to accommodate circadian effects across brain tissues.

## Countermeasures in use

The most prevalent and successful general countermeasure is exercise and the ISS offers a suite of different exercise devices including the Advanced Resistive Exercise Device (ARED), ergonomic bike (CEVIS) and treadmill (T2), combined these offer a full range of exercise modalities ranging from resistive exercise to aerobic. It remains to be known if the current combination of exercises is fully beneficial or if some elements lead to the development or aggravation of SANS. Indeed, it has been hypothesized that resistive exercise may aggravate SANS in part due to an associated increase in intrathoracic pressure which would translate to the brain and cause spikes in ICP. This notion is in part supported by the increase in incidence of SANS following deployment of the ARED, even though the first SANS case was seen in 2005 and ARED came a few years later to the station. Claims were further made that a synergistic effect between headward fluid shift, elevated CO_2_, and strenuous exercise created a perfect storm leading to SANS. However, both parabolic flights and ground-based trials including direct recording of ICP during exercise with and without elevated CO_2_ have demonstrated no associated pathological elevation in ICP^22^.

Lower body negative pressure (LBNP) has been shown to unload cerebral structures following a non-linear dose-response relationship and low-level^22,30^. Additionally, long-term, low-pressure LBNP (8 h per day during 3-day bedrest) has proven to ameliorate early signs of SANS^36^ and has been studied in long-term bedrest at the German Aerospace Center (DLR) :envihab facility. Long-term, low-level LBNP is currently the most promising countermeasure for SANS.

Investigations of the efficacy of systematic application of LBNP during spaceflight are warranted and required to determine an appropriate countermeasure regime (i.e., What is the optimal duration of application? Should LBNP be applied every day, several times per week, or on a monthly basis? Should LBNP be applied to all as a preventive measure (prophylactic) or only after detection of signs of SANS and used as a treatment?). Additionally, clarification of potential adverse effects such as cerebral hypoperfusion and ways of reducing this risk are needed. Although there have been no documented cases, there is, in theory, an elevated risk of DVT; the risk should be clarified and mitigated if warranted.

Venous-constrictive thigh cuffs are used by cosmonauts to ameliorate the symptoms of “head congestion” associated with cephalad fluid shift. In-flight experimental data of short-term (10–30 min) inflation of thigh cuffs have demonstrated increased volume of the legs along with 20% reduction of internal jugular venous diameter, thus indicating the desired caudal fluid shift along with reduction in facial edema. However, ground-based studies directly measuring ICP during thigh cuff inflation have not indicated significant reductions in ICP.

It is well known that an Impedance Threshold Device (ITD) reduces central venous pressure and this has been utilized clinically to augment venous return and cardiac filling. Ground-based studies have demonstrated that ITDs convergingly lowers ICP; however, feasibility and comfort of wearing a tight-fitting face mask or breathing through a mouthpiece for several hours per day may pose a challenge for crew compliance.

Bed rest studies with strict head-down tilt showed signs of SANS such as optic disc edema and choroidal and retinal folds^37^. Different countermeasure methods had been applied during bed rest studies to prevent SANS. One of the most important and promising countermeasures seems to be the lifting of the head. During strict head-down tilt, application of 1 g artificial gravity of 30–60 min every day does not seem to prevent SANS.

Previous HDBR experiments have shown that thigh cuffs limit the symptoms due to fluid shift and the loss in plasma volume in HDBR. They reduced jugular cross-section and frontal head edema but did not counteract orthostatic intolerance. Thigh cuffs have also been applied during −6° head-down tilt bed rest during a NASA SANS Countermeasure study at DLR and do not seem to prevent optic disc edema.

Dry immersion is a method that allows for a rapid cardiac deconditioning and concurrent fluid migration toward the upper body. Kermorgant et al.^38^ showed that a dry immersion of 3 days resulted in a rapid and persistent increase in optic nerve sheath diameter (ONSD). These changes were observed much more rapidly than in an HDBR experiment. A comparison of the effects of dry immersion for brain and eye with bed rest should be addressed in the near future. The isolated circulatory effects of cardiac deconditioning for fluid turnover in the head may be studied with DI very well.

A ground simulation of microgravity, using the dry immersion (DI) model (5 days), was performed to assess the effects of thigh cuffs (worn 10 h/day) on body fluid changes and dynamics, as well as on cardiovascular deconditioning. Thigh cuff countermeasure slowed down and limited the loss of body water and tended to limit plasma loss induced by DI but did not counteract decreased tolerance to orthostatic challenge. In this experiment DI provoked a slight but significant increase in retinal nerve fiber layer thickness (RNFLT) in the temporal quadrant and an enlargement in ONSD. Thigh cuffs tended to limit the ONSD enlargement but failed to prevent the increase in RNFLT^38,39^.

Novel neuroimaging techniques, such heavily weighted T2-FLAIR after intravenous administration of gadolinium-based contrast material may be useful^40^. The latter may be useful for the study of glymphatics in the optic nerve and in the CNS for ground analog experiments and for astronauts preflight and post-flight.

Low dose radiation, social isolation, and cardiovascular changes such as endothelial injury, central venous pressure changes, type and intensity of exercise, environmental factors such as the ambient air, nutrition including vitamins, and genetic susceptibility may contribute to changes related to SANS and they need to be considered as confounding factors.

## KEY KNOWLEDGE GAPS

While the exact etiology of SANS remains undetermined, biomarkers to monitor SANS, and countermeasures to mitigate and treat SANS are being developed and tested in ground analog environments and in preliminary in-flight trials. Implementation of countermeasures is timely as the consequences of SANS may be critical both for astronaut performance and health and mission success. Steps to understand underpinning mechanisms should be taken immediately because they inform biologically plausible biomarkers to monitor and countermeasures to mitigate and treat SANS. Importantly, the successful outcome of a countermeasure to either prevent or treat SANS will provide information about the underlying (patho)physiological processes and iterative improvement of measurements.

An overarching issue is the inherently small sample size. It is recommended to include predefined biomarkers, and eye and brain measurements, as part of core data / standard objective measurements in human ground analog studies, and make these available to the community for broader analyses in larger samples. While standard measures pre- and post- analog and in-flight have already been incorporated, there is a lack of long-term follow-up standard measures and definition of minimal follow-up periods. Therefore, the incorporation of longer-term follow-up studies is recommended to determine whether the eye and brain changes are reversible.

## Biomarkers

Biomarkers can be imaging-based, biochemical from body fluids, or functional. They should be non-invasive, reliable, and practical so they can be performed before, during, and after flight. Measurements across crewmembers (timeline) both in-flight and during analogs should be standardized along with systematic disclosure of the measurement conditions. For relevant eye and brain parameters that are not directly measurable in-flight, the development and validation of surrogate biomarkers should be pursued. Measurements of parameters related to eye and brain changes at multiple time points – ideally continuously – and simultaneously would enable the creation of comprehensive and personalized time-series data sets of each participant astronaut. The increased temporal sampling will improve the statistical analysis approaches of these dynamic measurements even in light of small cohorts, rather than relying on single data points or age-matched normative database comparisons.

Currently, most of the biomarkers used to monitor eye and brain changes are based on the structural changes assessed by imaging modalities such as optical coherence tomography (OCT) and MR. While visual acuity in distance and near is always tested pre-, in-, and post-flight, visual acuity under reduced contrast, eye ultrasound, OCT, fundus imaging, and tonometry can also be performed pre-, in- and post-flight. As MR can be performed only on the ground before and after the flights, the time course of the brain changes in flight is not yet known. In addition, biochemical biomarkers from body fluids other than blood (e.g., tear fluid, saliva, urine) or functional biomarkers for SANS are lacking. In that regard, a pilot study on blood-based biomarkers for brain-structural integrity already showed its usefulness by uncovering substantial signs of brain injury in long-duration cosmonauts^41^.

Visual performance in the challenging environment of space is not well understood, but may have a major impact on mission success, especially in missions longer than 1 year. Currently, high-quality visual function tests are not performed on astronauts. Vision function changes need to be monitored using technology adapted to in-flight use and reported along with other sensory and behavioral changes. The eye changes in the scope of SANS require in-depth investigations. An uneven hyperopic shift of >1 diopter^3^ can create anisometropia and reduce stereovision, an important cue in space to correctly estimate distances and locate objects, and operate heavy machinery. Optic disc edema can change color perception, and anomaloscopy can quantitatively identify these changes. The paucity of reliable and non-invasive biomarkers may delay progress in the risk assessment and development of optimal countermeasures.

Technological advances and capacity building for biomarkers are needed: The development of a small non-contact IOP measurement device, 3D-ultrasonography^42^ enhanced by hybrid techniques such as multispectral photoacoustic tomography^43^, and near-infrared hyperspectral imaging of the eye^44^ for structural and functional imaging during spaceflight would be very helpful.

The risk assessment for the development of SANS, or progression of brain-structural alterations, and the outcomes of current or potential countermeasures should be monitored with biomarkers as recommended by regulatory agencies such as the European Medicines Agency (EMA) and the US Food and Drug Administration (FDA). Biomarkers are defined as characteristics that are measured as an indicator of normal biological processes, pathogenic processes, or responses to an exposure or intervention, including therapeutic interventions^45^.

Development of biochemical body fluid biomarkers, and metabolic and functional eye and brain imaging modalities would be helpful for structural-functional correlation at earlier stages. This could be a link between the function/cognitive/performance element and structural hardcore changes in SANS.

We suggest a more integrative approach in the study of SANS with closer connection between eye and CNS changes. For volume/pressure changes of CSF, in addition to intracranial CSF, changes in the spinal CSF compartment should be taken into account as elongation of the spine in-flight^46^ may initially extend the CSF reserve capacity and influence CSF dynamics. These studies should also include the relation between eye and brain changes and body parameters.

Measurements of the relevant parameters should be performed just before and right after re-entry and on subsequent days to assess rapid (re)adaptive processes after return to Earth. Systematic study of the long-term course of the structural and functional effects on the brain and eye would be essential to determine whether changes are fully reversible possibly due to “passive hydrostatic/gravitational” effects, or irreversible which may indicate that there is an underlying and ongoing pathological process.

## Countermeasure

The countermeasure strategies that are currently used, under validation or consideration can be divided into systemic and local interventions. Systemic countermeasures include exercise, artificial gravity (integrative and systematic) by spacecraft rotation or by short arm centrifuge, LBNP, and pharmacological countermeasures that target SANS, cardiovascular changes, and radiation effects. Local countermeasures may have the advantage to prevent or treat SANS, without affecting the systemic adaptive processes. They may include the modulation of IOP by topical pharmacology approaches and the mechanical devices such as pressurized goggles with the risk of aggravating the SANS condition^47^. This approach would need much further research as it could have negative impacts on the optic nerve head and its structures. For example, the effects of the intensity or dose, duration and timing (relative to circadian rhythm and the flight) of different modalities of exercise or other countermeasures on SANS are only partially known.

It appears likely now that no individual countermeasure alone will completely prevent SANS, but that a combination of various countermeasures will be needed.

The safety and efficacy of a single or a combination of countermeasures should be tested in studies similar to small-sample size clinical trials with statisticians as an integral part of the team designing the study.

The effects of the intensity or dose, duration, and timing (during the circadian rhythm and relative to the flight) of a countermeasure such as exercise on SANS are only partially known. These parameters along with body weight, anthropometric measurements, BMI, gender and age, should be taken into account in design and analysis, and they should be disclosed with the characteristics of the participants in a systematic manner in peer-reviewed publications and in open source databases after publication.

## Analogs

**Fig. 1:**
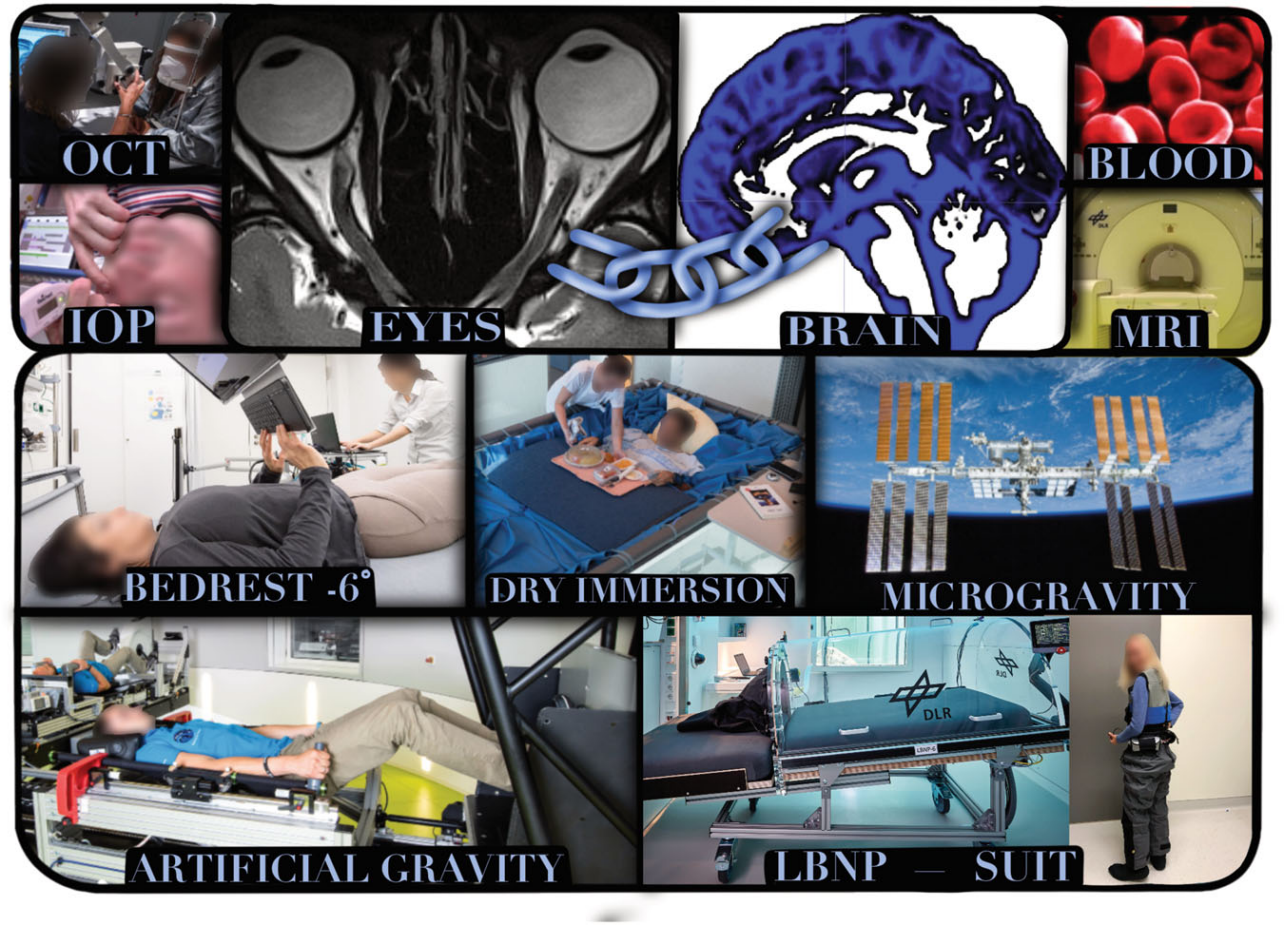
Eyeand brain examinations and possible countermeasures in space and analogs contributing to the understanding of SANS. It is crucial to adopt a comprehensive perspective that integrates eye and brain observations and analysis when approaching SANS. Optical coherence tomography (OCT) and magnetic resonance imaging (MRI) are performed together with tonometry to measure intraocular pressure (IOP), the main working horses in detecting eye and brain changes. A –6° head-down tilt bedrest and dry immersion are excellent analogs to simulate the effects of microgravity on the human body. Artificial gravity and lower body negative pressure (LBNP) are both investigated aspotential countermeasures to mitigate the effects of fluid shift and increased cerebral blood flow. Credit: Figure by Johanne AG Petersen, images modified from ESA- and DLR-web portal.

The term BANS – bedrest associated neuro-ocular syndrome – is used to describe the different but similar phenomena of SANS-like changes during head-down tilt bed rest (Table 1). It is not clear so far how the physiology challenge from ground analogs can be translated to inflight. Laurie, et al., found that peripapillary total retinal thickness increased to a greater degree among 11 bed rest test subjects than among 20 astronauts^37^. Conversely, choroid thickness did not increase among the individuals exposed to bed rest but increased among the astronauts^37^. Standard examinations at standard intervals in all different analogs will allow comparing the effects of the different analogs on the brain and eyes and give potentially more insight into the etiology of SANS (Fig. 1).

ISS will be used as an analog for the Moon in giving the astronauts more training and autonomy in the examinations. The Moon and the Lunar Gateway will be used as Mars analogs in building and using small and light instruments for important and necessary examinations. Astronauts will be trained in autonomous health care decision making.

**Fig. 2:**
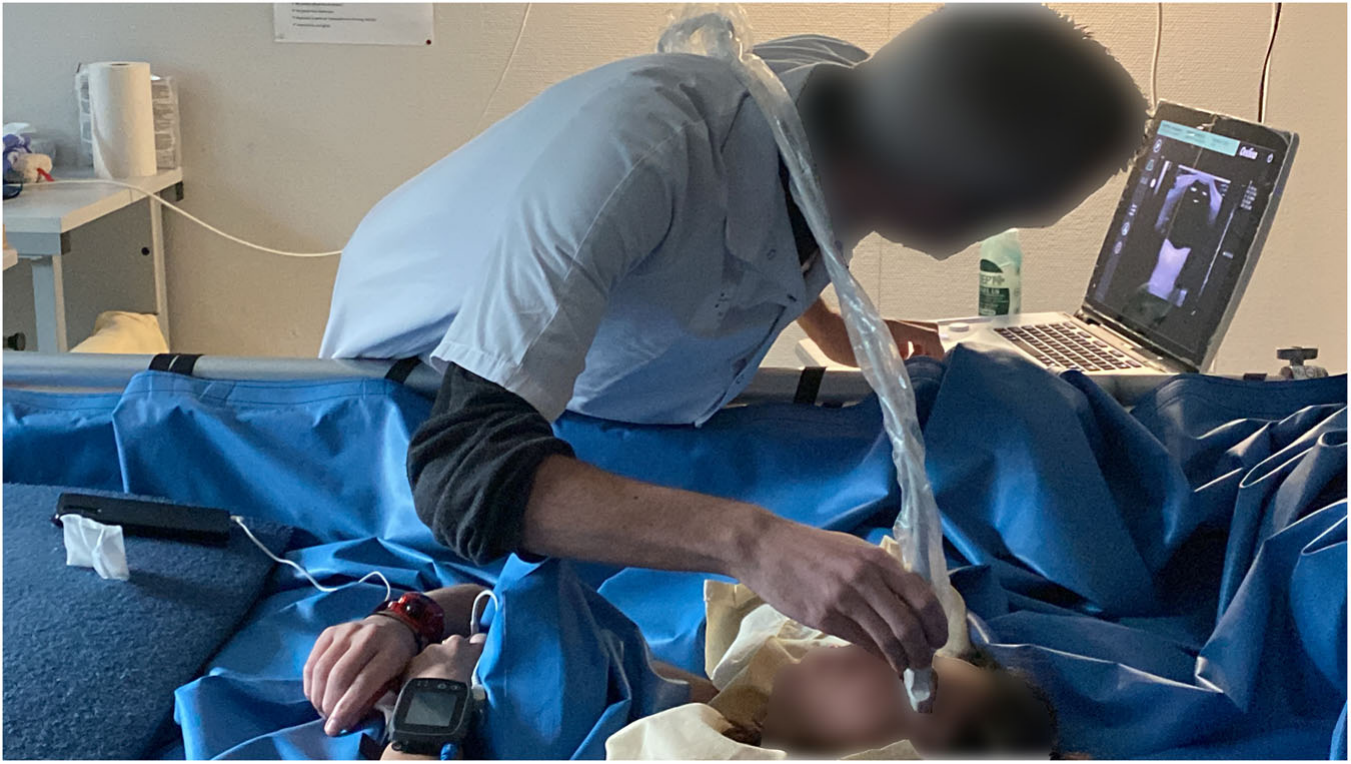
Optic Nerve Sheath Diameter measurement by ultrasound in Dry Immersion (Credit: MEDES).

Bed rest studies with more frequent OCT and fundus examinations should be performed (day 2, 3, 4, 5, 7, 10, 15, 20, 25, 30). OCT examinations should include the OCT international standard measurements for optimal comparison. OCT anatomical positioning system (APS) and angiography give even more information about the optic nerve head and vessel changes; these examinations are already in place in bedrest studies. The same ophthalmological measurements are performed in dry immersion studies (Fig. 2). Core sMRI should be performed in studies >14 days every 10 days and at R + 0.

Other analogs, such as social isolation and confinement, may help to determine possible confounding and/or synergistic factors contributing to SANS. Specifically, stress and disturbed sleep may aggravate symptoms.

Experimental animal models may be useful to understand the effect of headward fluid shift on the eye and the brain. Experiments in nonhuman primates such as continuous IOP and ICP measurements during posture changes^16^ are helpful to understanding their interplay, and the development and validation of new non-invasive tools to measure them in astronauts.

Rodent models present some advantages, such as the presence of the mouse facility at ISS^48,49^, due to their small size, similarity in vascular, brain, and eye physiology to humans, and the availability of the transgenic mice that lacks the critical molecular pathways involved in the fluid transports. Furthermore, the adaptation of NASA’s headward fluid by hindlimb unloading in rodents^50^ adapted for the study of SANS may help to understand the involved fundamental mechanisms and the possible interactions between environment (headward fluid shift and/or radiation)^51^, genotype (critical molecular pathways), and time (duration of headward fluid shift) in the development, progression and prevention of SANS.

The study using ground analogs should be integrated with research and development partnerships and efforts to develop new technologies to monitor and mitigate SANS-related eye and brain changes.

As substantial gaps remain in scientific knowledge about SANS, the space health community may recommend a minimum set of standardized tests to assess the eye and brain changes during ground analog studies performed for other health risks and their countermeasures (Table 2).

**Table 2:** Eye and brain roadmap recommendations.

1. MECHANISM
1.1. Understand underpinning mechanisms to inform biologically plausible biomarkers to monitor, mitigate and treat SANS.
2. BIOMARKERS
2.1. Include predefined biomarkers (e.g., imaging-based, biochemical from body fluids, or functional), and eye and brain measurements, as part of core data / standard objective measurements in human ground analog studies.
2.2. Make these predefined biomarkers available to the community for broader analyses in larger samples.
2.3. Incorporate these biomarkers into longer-term follow-up studies to determine whether the eye and brain changes are reversible.
2.4. Obtain non-invasive, reliable, and practical biochemical biomarkers from body fluids other than blood (e.g., tear fluid, saliva, urine), imaging and functional biomarkers.
2.5. Develop biochemical body fluid biomarkers, and metabolic and functional eye and brain imaging modalities to enable structural-functional correlation at earlier stages.
3. EXAMINATIONS / TESTING / MEASUREMENT
3.1. Obtain high-quality visual function tests from astronauts during their missions.
3.2. Perform OCT and fundus examinations during bed rest studies with more frequency (day 2, 3, 4, 5, 7, 10, 15, 20, 25, 30). In-flight OCT examinations should include the OCT international standard measurements for optimal comparison, OCT anatomical positioning system (APS), and angiography for more information about the optic nerve head and vessel changes. Core sMRI should be performed in studies > 14 days every 10 days and at R + 0.
3.3. Obtain measurements of parameters related to eye and brain changes at multiple time points – ideally continuously for 24 h – and simultaneously to enable the creation of comprehensive and personalized time-series data sets of each participant astronaut.
3.4. Take measurements of the relevant parameters just before and right after re-entry and on subsequent days to assess rapid (re)adaptive processes after return to Earth to determine the extent to which changes are reversible or irreversible.
3.5. Develop an integrative approach to study SANS with closer connection between eye and CNS changes (e.g., consider changes in the spinal CSF compartment and body mass).
4. ANALOGS / MODELS
4.1. Improve understanding of how the physiology challenges from ground analogs could be translated to inflight by creating standard examinations at standard intervals in all different analogs to accurately compare the effects of the different analogs on the brain, eyes, and etiology of SANS.
4.2. Use experimental animal models (e.g., nonhuman primates, rodents) to better understand the effect of the environment (headward fluid shift), genotype, and time duration of exposure on the eye and the brain and the development, progression, and prevention of SANS.
4.3. Standardize measurements across crewmembers (timeline) both in-flight and during analogs, along with systematic disclosure of the measurement conditions. For relevant eye and brain parameters that are not directly measurable in-flight, develop and validate surrogate biomarkers.
4.4. Integrate ground analog studies with research and development partnerships and efforts to develop new technologies to monitor and mitigate SANS-related eye and brain changes.
4.5. Develop a minimum set of standardized tests to assess the eye and brain changes during ground analog studies performed for other health risks and their countermeasures.
4.6. Perform social isolation, confinement, stress, and disturbed sleep analogs to determine possible confounding and/or synergistic factors contributing to SANS.
5. TECHNOLOGY
5.1. Develop a non-contact IOP measurement device, 3D-ultrasonography enhanced by hybrid techniques (e.g., multispectral photoacoustic tomography), and near-infrared hyperspectral imaging of the eye for structural and functional imaging during spaceflight.
5.2. Develop technology adapted for in-flight use to monitor vision function changes and other sensor and behavioral changes.
6. COUNTERMEASURES
6.1. Understand the effects of intensity or dose, duration and timing (relative to circadian rhythm and the flight) of different modalities of exercise or other countermeasures on SANS.
6.2. Consider intensity of dose, duration, and timing of a countermeasure along with body weight, anthropometric measurements, BMI, gender, and age in study design and analysis.
6.3. Test the safety and efficacy of a single or a combination of countermeasures in small-sample size clinical trials with statisticians as an integral part of the team designing the study.

Recommendations of the European Expert Group for future testing, evaluating and researching for a better understanding of SANS.

### Reporting summary

Further information on research design is available in the Nature Research Reporting Summary linked to this article.

## Supplementary information

Reporting Summary

## Data Availability

Data sharing is not applicable to this article as no new data were created or analyzed in this study.
